# The Psychosocial Pathways of Expectancy-Value Beliefs, Physical Fitness, and Executive Function Among Adolescents

**DOI:** 10.3390/healthcare14193305

**Published:** 2026-10-03

**Authors:** Dijun Shen, Sarina Bao, Xiaoxia Zhang

**Affiliations:** 1 Department of Physical Education, Shanghai Lixin University Accounting and Finance, Shanghai 201620, China; shendijun1988@163.com; 2 Department of Earth and Planetary Science, Graduate School of Science, The University of Tokyo, Tokyo 113-0033, Japan; bao-sarina1111@g.ecc.u-tokyo.ac.jp; 3 School of Sport Sciences, West Virginia University, Morgantown, WV 26506-6116, USA

**Keywords:** adolescents, executive function, physical fitness, expectancy-value beliefs, cognitive health, psychosocial mechanisms, structural equation modeling

## Abstract

**Highlights:**

**What are the main findings?**

- Adolescents with stronger expectancy-value beliefs showed higher levels of physical fitness.
- Physical fitness significantly mediated the association between expectancy-value beliefs and executive function.

**What are the implications of the main findings?**

- The findings support a psychosocial pathway linking motivation, physical fitness, and cognitive health.
- Future experimental intervention research can further test if strengthening perceived competence and task values may contribute to better physical fitness and executive function in adolescents.

**Abstract:**

**Background/Objectives**: Adequate physical fitness has been associated with cognitive health among adolescents, and psychosocial processes may help explain this relationship. However, evidence regarding the mechanisms linking physical fitness and cognitive health remains limited. Guided by expectancy-value theory, this study examined the direct and indirect effects of expectancy-value beliefs on executive function through physical fitness among adolescents. **Methods**: A prospective research design was used. Participants were 424 adolescents (58% female; Mage = 12.72 years, SD = 0.93) recruited from the southwestern United States. Physical fitness components, including cardiovascular fitness, muscular fitness, flexibility, and body mass index (BMI), were assessed using the FITNESSGRAM^®^ test battery. Expectancy-value beliefs and executive function were measured using validated self-report instruments. Structural equation modeling was conducted to examine the proposed relationships. **Results**: Expectancy-value beliefs were significantly associated with all physical fitness components (rs ranged from −0.86 to 0.46, ps < 0.01) except flexibility. Cardiovascular fitness and muscular fitness were negatively associated with executive function scores (rs ranged from −0.29 to −0.21, ps < 0.01), whereas BMI was positively associated with executive function scores. The proposed model demonstrated an acceptable fit (χ^2^/df = 284.16/113, *p* < 0.001; CFI = 0.90; RMSEA = 0.06, 90% CI [0.05, 0.07]). There was a significant indirect effect of expectancy-value beliefs on executive function through physical fitness (β = −0.11). The model explained 12% of the variance in physical fitness and 9% of the variance in executive function. **Conclusions**: The findings provide evidence for the proposed psychosocial mediation model linking expectancy-value beliefs, physical fitness, and executive function among adolescents. It suggests strategies that strengthen perceived competence and task values may improve physical fitness and, in turn, enhance executive function.

## 1. Introduction

Adolescence is a developmentally sensitive period marked by rapid neurological maturation, physical growth, and psychosocial change [1]. Against this backdrop, two interrelated public health concerns have drawn sustained research attention: the rising prevalence of childhood overweight and obesity, and the growing burden of mental health conditions among school-aged youth. In the United States, obesity affects approximately 19.7% of children and adolescents aged 2–19 years [2], with more recent surveillance data from the Centers for Disease Control and Prevention reporting a prevalence of 21.1% during 2021–2023 [3]. According to the World Health Organization [1], more than 390 million children and adolescents aged 5–19 years were overweight or obese in 2022, rising from 8% in 1990 to 20% within three decades. As documented by the GBD 2021 Adolescent BMI Collaborators [4], if current trajectories continue, approximately one-third of the world’s children and adolescents will be overweight or obese by 2050.

Concurrently, mental health disorders represent a significant and underappreciated burden in youth. Approximately 20% of children and adolescents in the United States meet criteria for a diagnosable mental disorder [5]. More recently, research suggests that physical inactivity and depression are co-occurring at alarming rates among this age group [6]. These disorders disrupt cognitive regulation, interpersonal functioning, and educational attainment, highlighting the urgent need to identify modifiable factors that support cognitive health during adolescence. Among these factors, health-related physical fitness (PF) has attracted growing scientific interest as a potentially powerful leverage point for school-based intervention.

It was well-documented that higher levels of health-related PF, encompassing cardiovascular fitness, muscular fitness, flexibility, and body composition, are positively associated with cognitive function and academic performance in youth [7,8,9]. Donnelly et al. [7], in a comprehensive systematic review, concluded that positive associations exist among physical activity, fitness, and cognitive outcomes in school-aged children, with particular evidence implicating cardiorespiratory fitness (CRF) as a driver of executive functioning. This relationship appears to operate through neurobiological mechanisms; that is, regular aerobic exercise induces short- and long-term structural and functional changes in brain regions involved in learning and memory, including increased cerebral blood flow, neurogenesis, and elevated brain-derived neurotrophic factor (BDNF) levels [10,11]. Cross-sectional studies have further shown that higher CRF and more favorable body composition profiles are independently associated with better executive function performance across multiple cognitive domains in adolescents [12,13].

Executive function (EF), defined as the set of top-down cognitive control processes encompassing inhibitory control, working memory, and cognitive flexibility, represents one of the most clinically relevant developmental outcomes during adolescence [14]. As a functional construct, EF governs the capacity to resist impulsive behavior, sustain goal-directed attention, and organize purposeful action [14]. Executive dysfunction is implicated in ADHD, depression, anxiety, and poor academic achievement [15], making it a priority target for health-oriented school programming. A systematic review and meta-analysis confirmed that both acute and chronic physical exercise yield significant positive effects on multiple EF domains in adolescents, including inhibitory control, working memory, and cognitive flexibility [16]. Importantly, the type, intensity, and duration of physical activity modulate the magnitude of these cognitive benefits, pointing toward the need to understand the contextual conditions, including motivational processes, under which fitness-EF relationships are most robust [17,18].

Although the neurobiological pathways connecting physical fitness to cognition are well established [19], emerging structural equation modeling (SEM) research has begun to illuminate physical fitness (PF) as a critical mediating variable within more complex biopsychosocial models. Lemes et al. [20] demonstrated that physical fitness, encompassing cardiorespiratory fitness, muscular fitness, and speed-agility, significantly mediated the relationships among personal, social, lifestyle, and cognitive performance factors in a large Latin American adolescent sample. Similarly, Bao et al. [21] showed that cardiorespiratory fitness and muscular fitness significantly mediated the association between moderate-to-vigorous physical activity and executive functions in primary school children, whereas motor competence did not exhibit a significant mediating effect. Consistent with this evidence, a recent scoping review by Marrero-Rivera et al. [22] reported that the majority of studies in school-aged Latino children found positive associations between physical fitness and cognitive outcomes, while also highlighting a substantial gap in clarifying the mechanisms underlying these relationships. The psychosocial mechanisms through which physical fitness contributes to executive functioning remain poorly understood, particularly within integrated models incorporating motivational and psychosocial determinants.

Expectancy-value theory (EVT) [23] offers a well-validated motivational framework for understanding students’ achievement-related behaviors in academic and physical activity contexts. EVT posits that motivation is shaped by two interrelated dimensions: (a) expectancy beliefs, subjective judgments about one’s competence and anticipated success on a given task; and (b) subjective task values, the perceived incentives for engaging in the task, including attainment value, intrinsic value, and utility value. Together, the EVT motivation predicts effort, persistence, and achievement behavior in a given domain [24,25].

A growing body of research has applied EVT to physical education (PE) settings, showing that students with higher expectancy beliefs and task values toward PE are more likely to achieve better physical fitness outcomes [26,27]. A meta-analysis of 31 EVT studies in PE [28] concluded that EVT motivation predicts a wide range of student outcomes, including physical fitness, in-class physical activity, health behavior, situational interest, and out-of-school participation. Within this body of work, Chen et al. [29] found that middle school students’ expectancy beliefs significantly predicted cardiorespiratory fitness and muscular strength and endurance, even after controlling for gender and weight status. Gu and Solmon [30] extended these findings to show that EVT-related motivational processes predicted psychosocial well-being among elementary children. Despite these advances, the motivational pathway from EVT motivation → physical fitness → executive function has not been empirically tested in an integrated model given the theoretical and practical relevance of this chain of effects.

A healthcare perspective further emphasizes the importance of psychosocial mechanisms in adolescent cognitive health. EVT motivation has been found to predict health outcomes among at-risk youth populations, including those from lower socioeconomic backgrounds and underrepresented ethnic groups [31,32]. Because health disparities in physical fitness, obesity, and mental health are well-documented across demographic groups, identifying modifiable psychosocial determinants, such as students’ expectancy beliefs and values, could inform equitable, scalable interventions. School PE programs represent particularly viable delivery vehicles, and they provide opportunities to build competence perceptions and fitness simultaneously [33,34]. Studying this psychosocial mechanism can facilitate further investigations on whether improving students’ expectancy beliefs and values could improve cognitive functioning, potentially narrow disparities in both physical and mental health.

Despite considerable evidence for the individual links between (a) EVT motivation and physical fitness, and (b) physical fitness and executive function, these pathways have rarely been studied in a single unified model and further tested with adolescents in a school context. The present study addresses this gap by testing an EVT-grounded psychosocial model in which expectancy-value beliefs predict executive function both directly and indirectly through health-related physical fitness among middle school adolescents. The first purpose of this study was to examine the associations among expectancy-value beliefs, health-related physical fitness components (cardiovascular fitness, muscular fitness, flexibility, BMI), and executive function indicators (behavioral regulation, metacognition). The second purpose was to examine whether health-related physical fitness mediates the effect of expectancy-value beliefs on executive function among adolescents. It is hypothesized that expectancy-value beliefs would directly predict executive function and indirectly predict executive function through health-related physical fitness. Findings are expected to advance theory and practice by clarifying a motivational pathway to cognitive health and informing the design of motivation-centered school- and community-based health interventions.

## 2. Materials and Methods

### 2.1. Participants

This study was conducted in three middle schools in the southwest region of the United States. A total of 424 adolescents (246 girls and 178 boys; Mage = 12.70, SD = 0.93) participated in this study. There were 38.2% in the 6th grade (n = 162), 36.1% in the 7th grade (n = 153), and 25.7% in the 8th grade (n = 109). More than half of participants were Caucasian (57.1%), and the remaining participants included 23.6% Hispanic, 10.2% Black, 4.3% Asian, and 4.8% of other ethnicities. The majority of participants (73.4%) were from high-SES families identified by each participant’s lunch meal status according to the Income Eligibility Guidelines (IEGs) [35].

This study was approved by the University Institutional Review Board (IRB code 14150, and date of approval 31 March 2014). Approval from the school district and school principals was also obtained prior to the beginning of the study. Participating children and their parents signed the informed consent form before data collection.

### 2.2. Research Design and Procedures

A prospective research design was used and conducted across one academic school year. In the fall semester, students completed a survey assessing their expectancy-value perceptions on physical education. Five months later, students’ health-related fitness was assessed using the FITNESSGRAM^®^ test battery [36]. At the end of the spring semester, students self-reported using the Behavior Rating Inventory of Executive Function-Self-Report form (BRIEF-SR [37]) in the classroom. Students were encouraged to answer truthfully and were assured that their responses would be anonymous and not affect their grades.

### 2.3. Measures

#### 2.3.1. Expectancy-Value Beliefs

A previously validated survey was used to evaluate students’ expectancy-value beliefs about physical education [24,38]. This self-report questionnaire is a 5-point Likert-type scale with five items measuring expectancy-related beliefs and six items measuring subjective task values. Expectancy-related beliefs were composed of students’ beliefs about their ability (three items) and expectancies about their success (two items). The example questions for beliefs about ability include (a) How good are you at activities and games in P.E.? (1 = very bad, 5 = very good); and (b) If you were to list all the students in your P.E. class from the worst to the best, where would you put yourself in? (1 = very bad, 5 = very good). The questions for expectancies on success are: (a) How good would you be at learning something new in P.E. this year? (1 = very bad, 5 = very good); and (b) How well do you think you will learn activities and games in P.E. this year? (1 = not at all well, 5 = very well). Cronbach’s alpha for this subscale is 0.73. Subjective task values consisted of students’ perceptions of the importance, interests, and utility of their P.E. Two questions addressed the component of attainment value or importance perception, such as “For me, being good at activities and games in P.E. is…” (1 = not very important, 5 = very important). Two questions evaluated the component of intrinsic or interest value. The example question is “In general, I find learning new activities and games in P.E. is… (1 = very boring, 5 = very fun)”. The component of utility value or usefulness was measured by two questions, such as “Compared to your other school subjects, how useful is what you learn in P.E.?” (1 = not useful at all, 5 = very useful). The Cronbach’s alpha for this subscale is 0.74. The whole scale showed good internal consistency with a Cronbach’s alpha value of 0.82. This scale has been documented as reliable and valid among the adolescent population [29,39].

#### 2.3.2. Health-Related Physical Fitness

FITNESSGRAM^®^ test battery [36] was utilized to measure participants’ PF. Four components of fitness were assessed, including body composition, cardiovascular fitness, muscular fitness, and flexibility. Specifically, students’ body mass index (BMI) was used to assess body composition. The BMI was calculated from the measured height and weight using the Health-o-Meter 500KL digital physician height and weight scale. Students’ cardiovascular fitness was assessed by the estimate of VO2max, which was predicted by participants’ age, gender, BMI, and number of successfully completed “laps” on the Progressive Aerobic Cardiovascular Endurance Run (PACER; [40]). abdominal and upper body muscular strength and endurance were used to measure muscular fitness. Abdominal strength and endurance were assessed by the number of completed curl-ups in a 4.5-inch curl-up test. Upper body muscular strength and endurance were tested by the number of completed push-ups in a 90° push-up test. Flexibility was measured by averaging the performance of students’ left and right legs on the sit-and-reach test. It has been indicated that these tests in the present study are reliable and valid to represent each component of health-related PF [41].

#### 2.3.3. Executive Function

The Behavior Rating Inventory of Executive Function, Self-Report (BRIEF-SR) [37] was applied to assess students’ executive function. BRIEF-SR is composed of 80 standardized items to assess 11–18-year-old adolescents’ executive functions through two broader indexes: the behavioral regulation index (BRI) and the metacognitive index (MI). BRI is indicated by the 4 non-overlapping subscales, including inhibit (13 items related to impulse control), shift (10 items related to the ability to move freely from one activity to another), emotional control (10 items related to the emotional response to a given situation), and monitor (5 items related to the awareness of his/her behaviors). MI is also indicated by 4 non-overlapping subscales. They are working memory (12 items related to the capability of holding information in mind for a given task or response), plan/organization (13 items related to the management ability for current and future-oriented activities or demands), organization of materials (7 items related to the orderliness of the everyday environment), and task completion (10 items related to the completion and perceived difficulties of a given task). All the subscales were rated on a 3-point Likert-type scale by responding to the frequency of a given situation: never, sometimes, and often (i.e., never means a desirable response and often means an impaired response). A higher score on BRI indicates the student has greater difficulty maintaining regulatory control of his or her behaviors and emotions. A higher score on MI indicates that the student has greater difficulty with problem-solving and organization in various contexts. The Cronbach’s alpha of this scale was 0.92, indicating sound internal consistency. Previous studies have revealed the reliability and validity of this scale when used in an adolescent population [42,43].

### 2.4. Data Analyses

Several steps of the data analysis were conducted using IBM SPSS Version 31.0 (IBM Corp., Armonk, NY, USA) for Windows. Prior to statistical analyses, the distribution, missing values, and outliers of the data were examined. Missingness across the primary variables ranged from 5% to just under 10%. Given that the volume of missing data was within acceptable thresholds [44], missing values were handled via a series of mean replacements. Cronbach alpha coefficient was used to assess the internal consistency of the survey variables (i.e., expectancy-value beliefs and executive function). The Pearson product-moment correlational analysis was used to examine relationships among variables.

Expectancy beliefs and subjective task values are theoretically distinguishable components within the expectancy-value framework [25]. In the present study, however, the research question concerns students’ overall motivational orientation toward PE rather than the differential prediction of outcomes from expectancies and values separately. We therefore modeled the construct as a single latent factor, which is also supported by the situated expectancy-value theory that expectancy beliefs and subjective task values represent theoretically distinct but related dimensions of achievement motivation [45,46]. We further conducted a principal axis factoring analysis to compare the one-factor and two-factor models. The results (not presented) revealed that the expectancy-value beliefs in the current sample had a better fit for one single dominant factor (eigenvalue of 3.936, explaining 35.78% of the total variance) rather than two dominant factors (factor 2 has an eigenvalue of 0.73, less than 1, explaining only 6.65% of the shared variance). Thus, in future analyses, we structured one latent factor for expectancy-value beliefs.

To further investigate whether physical fitness serves as a mediator in the relations between relationship expectancy-value beliefs (i.e., independent variable) and executive function (i.e., dependent variable), a structural equation modeling analysis (SEM) was conducted using Amos 31.0. Sample size adequacy was evaluated using commonly cited participant-to-estimated-parameter ratios in SEM. A re-examination of the AMOS output confirmed that the model contains 37 free parameters, including factor loadings, residual variances, factor variances, factor covariances, and structural paths. With n = 424, the participants-per-parameter ratio is approximately 11.5:1, which exceeds a conservative 10:1 guideline [47], indicating sufficient sample size for running the proposed model.

To start with, confirmatory factor analysis was conducted to evaluate the hypothesized measurement structure. In the structural model, the items of expectancy-value beliefs were treated as observed variables predicted by the latent variable (i.e., overall expectancy-value); the four components of health-related PF (i.e., BMI, cardiovascular fitness, muscular fitness, flexibility) were treated as observed variables predicted by the latent health-related PF; and the BRI and MI were treated as observed variables predicted by the overall executive function of participants. The model fit was tested by several goodness-of-fit statistics. The chi-square test (χ2) was applied to test the significant difference between the proposed model and the sample data, with the identified degrees of freedom (df). Other indices were the comparative fit index (CFI), normed fit index (NFI), incremental fit index (IFI), and root mean squared error of approximation (RMSEA). The values of CFI, NFI, and IFI range from 0 to 1. Each of these values greater than 0.90 refers to a good fit, and a value greater than 0.95 indicates an excellent fit [48]. The RMSEA has a value lower than 0.06, meaning a well-fit model [48], and a value between 0.06 and 0.08 indicating an acceptable model fit [49]. Lastly, bootstrapping with 2000 resamples was used to test the significance of the indirect effect of expectancy-value beliefs on executive function through physical fitness. Statistical significance was determined using a 95% bootstrap confidence interval (CI), with intervals excluding zero indicating a significant indirect effect.

## 3. Results

### 3.1. Descriptive Statistics and Correlations

Descriptive statistics for the total sample and by gender are presented in Table 1. Table 2 presents the bivariate correlations among all study variables. Expectancy beliefs were significantly and positively correlated with VO2max (r = 0.31, *p* < 0.01) and muscular fitness (r = 0.22, *p* < 0.01), and significantly and negatively correlated with BMI (r = −0.18, *p* < 0.01). Subjective task values showed a significant positive correlation with muscular fitness (r = 0.14, *p* < 0.05) but were not significantly associated with the other fitness components.

Regarding the relations between fitness and executive function, both VO2max (r = −0.29 for BRI; r = −0.21 for MI, both *p* < 0.01) and muscular fitness (r = −0.26 for BRI; r = −0.26 for MI, both *p* < 0.01) were negatively associated with both executive function indices, indicating that higher cardiovascular and muscular fitness were associated with better (lower-scored) executive function. BMI was positively correlated with BRI (r = 0.14, *p* < 0.05), meaning higher BMI was associated with poorer behavioral regulation. Flexibility was not significantly correlated with either BRI or MI. BRI and MI were strongly intercorrelated (r = 0.71, *p* < 0.01), consistent with the BRIEF-SR conceptual framework [36]. Expectancy beliefs were significantly and negatively correlated with BRI (r = −0.14, *p* < 0.05), suggesting that students with stronger expectancy beliefs demonstrated better behavioral regulation.

### 3.2. Structural Equation Modeling

To examine whether health-related physical fitness mediates the relationship between expectancy-value beliefs and executive function, a structural equation model (SEM) was tested using AMOS 31.0.

Prior to constructing the structural model, we conducted a measurement model with 11 expectancy-value items to further test construct validity according to the dimensional analysis results. The CFA revealed an acceptable fit: χ^2^/df = 119.65/40, NFI = 0.906, IFI = 0.935, CFI = 0.935, RMSEA = 0.069 (90% CI [0.051, 0.069]). Thus, the final model was structured with expectancy-value beliefs as the exogenous latent variable (with 11 observed indicators), health-related physical fitness as the mediating latent variable (with 4 observed indicators), and executive function as the endogenous latent variable (with 2 observed indicators).

The final SEM was acceptable: χ^2^/df = 284.16/113, NFI = 0.851, IFI = 0.904, CFI = 0.902, RMSEA = 0.060 (90% CI [0.051, 0.069]), at the borderline of well-fit thresholds of CFI and IFI ≥ 0.90 and RMSEA < 0.06 [48]. The variance explained by the model was 12% for physical fitness (R^2^ = 0.12) and 9% for executive function (R^2^ = 0.09). Full model fit indices and all standardized path coefficients are presented in Table 3.

**Figure 1:**
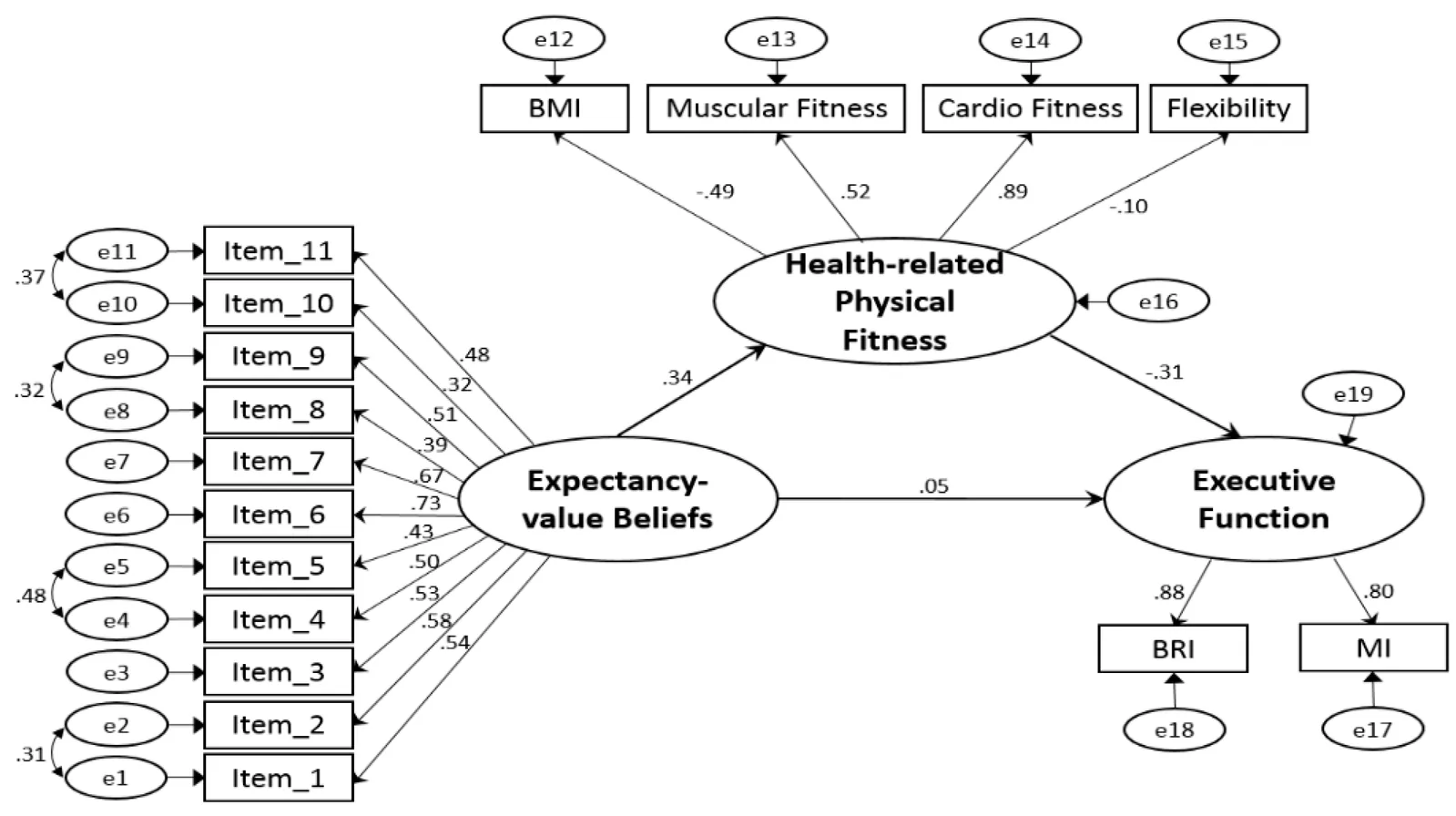
Structural equation model of expectancy-value beliefs predicting executive function through physical fitness. EF = executive function; BRI = behavioral regulation index; MI = metacognitive index; BMI = body mass index. The correlated residuals in expectancy-value beliefs’ observed indicators were theoretically adjusted: items 11 and 10 measure usefulness, items 9 and 8 measure interest value, items 5 and 4 measure success belief, and items 1 and 2 measure ability belief.

As shown in Table 3 and Figure 1, expectancy-value beliefs significantly and positively predicted health-related physical fitness (β = 0.342, CR = 4.353, *p* < 0.001), indicating that students with stronger expectancy beliefs and higher task values are associated with better overall physical fitness. Health-related physical fitness, in turn, significantly and negatively predicted executive function (β = −0.310, CR = −3.985, *p* < 0.001). Because the BRIEF-SR is scored such that higher scores indicate poorer executive function, this negative coefficient reflects a beneficial relationship: higher fitness was associated with better executive functioning. The direct path from expectancy-value beliefs to executive function was not statistically significant (β = 0.049, *p* = 0.508). The indirect effect of expectancy-value beliefs on executive function through physical fitness was significant (β = −0.106, bootstrapped 95% CI [−0.170, −0.048], *p* < 0.05). These results provide preliminary evidence supporting the full mediation of physical fitness in the expectancy-value beliefs to executive function pathway. That is, physical fitness statistically accounts for part of the association between expectancy-value beliefs and executive function scores.

Regarding the fitness indicators, VO2max was the strongest indicator of the physical fitness latent factor (β = 0.891, *p* < 0.001), followed by muscular fitness (β = 0.525, *p* < 0.001) and BMI (β = −0.489, *p* < 0.001). BMI is an indicator of body composition, and a higher BMI (especially when BMI is over 25) indicates a higher body fat percentage, meaning poor health-related fitness. Flexibility was not a significant indicator of the physical fitness latent factor (β = −0.098, *p* = 0.072), replicating the pattern observed in the bivariate correlations. For executive function, the BRI (β = 0.879, *p* < 0.001) and MI (β = 0.802, *p* < 0.001) were both strong indicators of the latent executive function construct.

## 4. Discussion

The purpose of this study was to examine how psychosocial motivational processes operationalized as expectancy-value beliefs interact with health-related physical fitness (PF) to influence cognitive health, specifically executive function (EF), among middle school adolescents. The findings support the hypothesized mediation model that expectancy-value motivation is associated with executive function indirectly through health-related physical fitness, while the direct association is non-significant. This study provided preliminary evidence of an integrated motivational-fitness-cognition model in a school physical education (PE) context. Three primary findings emerged and are discussed below.

Using a latent variable SEM approach, the present findings revealed that the relationship between expectancy-value beliefs and physical fitness is robust across multiple fitness indicators simultaneously. These results are supported by the psychosocial mechanism model proposed by Lubans et al. [11], which posits that psychosocial perceptions cultivated through physical activity participation, including ability beliefs and task values, shape motivational behaviors and physical fitness outcomes. This is also consistent with much previous research that when students believe they are capable and find PE important, interesting, and useful, they are more likely to exert effort and persist in activities benefiting their cardiovascular endurance, muscular strength, and body composition [24,25,28,29].

The pattern of full mediation provides strong empirical support for the psychosocial mechanism proposed by Lubans et al. [11], which hypothesizes that psychosocial perceptions arising from physical activity participation, such as expectancy beliefs and task values, enhance cognitive health outcomes by first improving physical fitness. The fitness-to-executive-function pathway is consistent with a well-established neurobiological literature demonstrating that higher levels of cardiovascular and muscular fitness are associated with better cognitive control among children and adolescents [7,8]. Physiologically, higher levels of physical fitness (e.g., being able to run longer, being able to lift heavier weights) are associated with more favorable neurobiological responses, including neuroplasticity, cerebral blood flow, and brain-derived neurotrophic factor (BDNF) levels, all of which support the neural substrates of executive function, including the prefrontal cortex and hippocampus [10,11]. The present SEM finding that VO2max was the strongest indicator of the fitness latent factor is consistent with prior evidence that cardiovascular fitness is the primary fitness dimension associated with cognitive outcomes [50,51].

Importantly, the Lubans et al. [11] psychosocial mechanism model distinguishes between neurobiological, behavioral, and psychosocial pathways. The present study specifically tests the psychosocial pathway: expectancy-value beliefs (a psychosocial construct) → physical fitness (the behavioral/physiological outcome of motivated engagement) → executive function (the cognitive health outcome). The non-significant direct path from expectancy-value beliefs to executive function further supports the interpretation that the motivational pathway to cognitive health operates indirectly, channeled through the students’ behavioral and physiological engagement in physical fitness. While the majority of the participants in this study were in high-SES status, future research should be cautious when interpreting these results for samples under different SES conditions.

The findings of this study carry meaningful implications for scholarly work in adolescent cognitive health. The psychosocial mechanism supported by the present study needs more future experimental intervention research to confirm the pathways to cognitive health: directly manipulating expectancy-value beliefs (e.g., through values-affirmation PE curricula or mastery climate interventions) and measuring downstream effects on both fitness and executive function. Given that executive function impairments are associated with ADHD, academic underperformance, and mental health problems in adolescence [15], future research may center on designing fitness- and motivation-focused PE programs and testing their effects on reducing executive dysfunction in school-aged youth. Future studies should also investigate potential moderators of the expectancy-value beliefs → fitness → executive function pathway, including gender, pubertal status, socioeconomic background, and specific PE instructional approaches.

Several limitations should be acknowledged. First, the prospective observational design of this study, with no baseline on executive function and fitness, prevents examining the changes over time; hence, no definitive causal inference can be concluded. Although the SEM tested a directional mediation pathway consistent with the Lubans et al. [11] framework, the model fit is borderline-to-acceptable, and future experimental or longitudinal designs are needed to confirm causal ordering. Second, executive function was measured via self-report (BRIEF-SR) rather than performance-based assessments (e.g., flanker task, N-back). Self-report EF may be subject to response biases, and future studies should incorporate neuropsychological performance measures. Third, the sample was drawn from three middle schools in the Southwest United States, limiting generalizability to other geographic regions, ethnic compositions, and grade levels. Future research should include more diverse samples, including racially and socioeconomically heterogeneous populations, as well as considering the potential clustering effects of school-level characteristics such as PE curriculum, resources, and teacher practices. Moreover, the measurement models for health-related physical fitness and executive function were not tested. It is known that health-related physical fitness is multidimensional in nature. BMI, cardiovascular fitness, muscular fitness, and flexibility are conceptually distinguishable components, consistent with the established theoretical framework of Caspersen et al. [52] and Ortega et al. [53]. Flexibility did not load significantly on the latent fitness factor, which is consistent with previous findings that flexibility shares relatively little variance with the other fitness components [54]. However, flexibility is a standard and theoretically integral component of health-related physical fitness in the literature [49,50]; thus, we retained it in the construct. The mathematical dependence between VO2max and BMI may have partially biased the loading; future studies should examine component-specific relations and consider alternative modeling approaches to address these dependencies. The measurement model for executive function, although not tested, consists of the behavioral regulation index and Metacognition Index that have been previously structured as observed indicators [55]. It is noted that the measurement model of the expectancy-value beliefs construct has model fit at the borderline-to-acceptable level, and a few indicators load below 0.50. While the measurement model is not the primary object of the study, readers can read Xiang et al. [38] for full psychometric detail. Lastly, while we had a modest proportion of missing data, using mean replacement may attenuate covariances, and future work would benefit from full information maximum likelihood (FIML) or multiple imputation.

## 5. Conclusions

This study provides preliminary evidence to support the psychosocial motivational pathway to adolescent executive function within a school PE context. That is, adolescents’ expectancy-value beliefs toward PE significantly predict their health-related physical fitness, which in turn significantly predicts their executive function. The indirect effect was significant while the direct effect was not, supporting full mediation and the key role of physical fitness as the bridge between psychosocial motivation and cognitive health. These findings shed light on the need for future PE-based experimental interventions to examine the effects of simultaneously cultivating adolescents’ competence beliefs and task values on both physical and cognitive health.

## Figures and Tables

**Table 1:** Descriptive statistics by gender.

Variable	Total (n = 424)	Boys (n = 178)	Girls (n = 246)
**Demographics**			
Age (years), M (SD)	12.72 (0.90)	12.48 (0.84)	12.89 (0.91)
Grade, n (%)			
6th grade	162 (38.2%)	—	—
7th grade	153 (36.1%)	—	—
8th grade	109 (25.7%)	—	—
Ethnicity, n (%)			
White	242 (57.1%)	—	—
Hispanic	100 (23.6%)	—	—
Black/African American	43 (10.2%)	—	—
Asian American	19 (4.3%)	—	—
Other	20 (4.8%)	—	—
**Study Variables, M (SD)**			
Expectancy Beliefs	4.18 (0.53)	4.21 (0.45)	4.16 (0.42)
Task Value	4.03 (0.56)	4.02 (0.45)	4.03 (0.48)
Health-related Physical Fitness			
Body Mass Index	21.36 (4.46)	20.46 (4.25)	22.06 (4.51)
Muscular Fitness (Push-ups)	15.73 (8.18)	17.63 (8.11)	14.26 (7.95)
Cardio Fitness (PACER laps)	36.93 (16.24)	42.60 (16.47)	32.58 (14.67)
Flexibility (Sit-and-Reach, inches)	12.52 (2.98)	11.11 (2.89)	13.58 (2.58)
Executive Function			
Behavioral Regulation Index	48.93 (11.03)	46.07 (9.32)	51.12 (11.74)
Metacognition Index	48.73 (12.40)	44.64 (10.25)	51.89 (13.00)

Note. M = mean; SD = standard deviation. Grade-level breakdowns by gender were not available in the coded dataset. Expectancy-value beliefs = mean composite of 11 survey items (scale: 1–5). Physical fitness indicators are from the FitnessGram^®^ battery. Executive Function scores are T-scores from the Behavior Rating Inventory of Executive Function (BRIEF).

**Table 2:** Descriptions and correlations among study variables.

Variable	1	2	3	4	5	6	7	8
1. VO2max (Cardiovascular Fitness)	—							
2. Muscular Fitness	0.42 **	—						
3. Flexibility	−0.26 **	−0.03	—					
4. Body Mass Index	−0.86 **	−0.26 **	0.21	—				
5. Behavioral Regulation Index	−0.29 **	−0.26 **	0.10	0.14 *	—			
6. Metacognition Index	−0.21 **	−0.26 **	0.12	0.12	0.71 **	—		
7. Subjective Task Values	0.11	0.14 *	0.12	−0.03	−0.10	−0.02	—	
8. Expectancy Beliefs	0.31 **	0.22 **	0.08	−0.18 **	−0.14 *	−0.04	0.54 **	—

Note. * *p* < 0.05. ** *p* < 0.01.

**Table 3:** Model fit indices and structural path coefficients of the SEM.

	χ^2^	df	NFI	IFI	CFI	RMSEA	90% CI [LL, UL]
**Model Fit Indices**							
Structural Model (SEM)	284.159	113	0.851	0.904	0.902	0.060	[0.051, 0.069]
**Structural Path Coefficients**							
Path (Predictor → Outcome)	B	SE	CR	β	*p*		
Direct Paths							
Expectancy-value Beliefs → Health-related Physical Fitness	14.811	3.402	4.353	0.342	***		
Health-related Physical Fitness → Executive Function	−0.235	0.059	−3.985	−0.310	***		
Expectancy-value Beliefs → Executive Function	1.594	2.410	0.661	0.049	0.508		
Indirect Path							
Expectancy-value Beliefs → Health-related Physical Fitness → Executive Function	—	—	—	−0.106 ^a^	***		

Note. For model fit: NFI = normed fit index; IFI = incremental fit index; CFI = comparative fit index; RMSEA = Root Mean Square Error of Approximation; 90% CI = 90% confidence interval for RMSEA. For structural paths: B = unstandardized coefficient; SE = standard error; CR = critical ratio; β = standardized coefficient. ^a^ Indirect effect is estimated as the product of standardized direct paths (β = 0.342 × −0.310 = −0.106); significance was evaluated using bootstrapping. *** *p* < 0.001.

## Data Availability

The data supporting the findings of this study are available from the corresponding author upon reasonable request. The data are not publicly available because they contain information that could compromise the privacy of research participants and are subject to ethical restrictions.

## References

[1] A World Health Organization Obesity and Overweight. https://www.who.int/news-room/fact-sheets/detail/obesity-and-overweight.

[2] Stierman B., Afful J., Carroll M.D., Chen T.C., Davy O., Fink S., Fryar C.D., Gu Q., Hales C.M., Hughes J.P. (2021). National Health and Nutrition Examination Survey 2017–March 2020 Prepandemic Data Files: Development of Files and Prevalence Estimates for Selected Health Outcomes. Natl. Health Stat. Rep..

[3] Centers for Disease Control and Prevention (2024). QuickStats: Prevalence of Obesity and Severe Obesity among Persons Aged 2–19 Years—United States, 1999–2000 through 2021–2023. MMWR Morb. Mortal. Wkly. Rep..

[4] GBD 2021 Adolescent BMI Collaborators (2025). Global, Regional, and National Prevalence of Child and Adolescent Overweight and Obesity, 1990–2021, with Forecasts to 2050: A Forecasting Study for the Global Burden of Disease Study 2021. Lancet.

[5] Merikangas K.R., He J.P., Burstein M., Swanson S.A., Avenevoli S., Cui L., Benjet C., Georgiades K., Swendsen J. (2010). Lifetime Prevalence of Mental Disorders in U.S. Adolescents: Results from the National Comorbidity Survey Replication–Adolescent Supplement (NCS-A). J. Am. Acad. Child Adolesc. Psychiatry.

[6] Rodriguez-Ayllon M., Cadenas-Sánchez C., Estévez-López F., Muñoz N.E., Mora-Gonzalez J., Migueles J.H., Molina-García P., Henriksson H., Mena-Molina A., Martínez-Vizcaíno V. (2019). Role of Physical Activity and Sedentary Behavior in the Mental Health of Preschoolers, Children and Adolescents: A Systematic Review and Meta-Analysis. Sports Med..

[7] Donnelly J.E., Hillman C.H., Castelli D., Etnier J.L., Lee S., Tomporowski P., Lambourne K., Szabo-Reed A.N. (2016). Physical Activity, Fitness, Cognitive Function, and Academic Achievement in Children: A Systematic Review. Med. Sci. Sports Exerc..

[8] Hillman C.H., Biggan J.R. (2017). A Review of Childhood Physical Activity, Brain, and Cognition: Perspectives on the Future. Pediatr. Exerc. Sci..

[9] Chacón-Cuberos R., Zurita-Ortega F., Ramírez-Granizo I., Castro-Sánchez M. (2020). Physical Activity and Academic Performance in Children and Preadolescents: A Systematic Review. Apunt. Educ. Fís. Deportes.

[10] Etnier J.L., Salazar W., Landers D.M., Petruzzello S.J., Han M., Nowell P. (1997). The Influence of Physical Fitness and Exercise upon Cognitive Functioning: A Meta-Analysis. J. Sport Exerc. Psychol..

[11] Lubans D., Richards J., Hillman C., Faulkner G., Beauchamp M., Nilsson M., Kelly P., Smith J., Raine L., Biddle S. (2016). Physical Activity for Cognitive and Mental Health in Youth: A Systematic Review of Mechanisms. Pediatrics.

[12] Cabral L.L.P., Browne R.A.V., Freire Y.A., Schwade D., Souto G.C., Dantas M., Lima F.A.S., Farias-Junior L.F., Costa E.C., Barros J.F. (2021). Cardiorespiratory Fitness and Performance in Multiple Domains of Executive Functions in School-Aged Adolescents. Front. Physiol..

[13] Meo S.A., Altuwaym A.A., Alfallaj R.M., Alduraibi K.A., Alhamoudi A.M., Alghamdi S.M., Akram A. (2019). Effect of Obesity on Cognitive Function among School Adolescents: A Cross-Sectional Study. Obes. Facts.

[14] Diamond A. (2013). Executive Functions. Annu. Rev. Psychol..

[15] Willcutt E.G., Doyle A.E., Nigg J.T., Faraone S.V., Pennington B.F. (2005). Validity of the Executive Function Theory of Attention-Deficit/Hyperactivity Disorder: A Meta-Analytic Review. Biol. Psychiatry.

[16] Xue Y., Yang Y., Huang T. (2019). Effects of Chronic Exercise Interventions on Executive Function among Children and Adolescents: A Systematic Review with Meta-Analysis. Br. J. Sports Med..

[17] Gu X., Zhang T., Chen S., Keller M.J., Zhang X. (2020). School-Based Sedentary Behavior, Physical Activity, and Health-Related Outcomes among Hispanic Children in the United States: A Cross-Sectional Study. Int. J. Environ. Res. Public Health.

[18] Zhang X., Gu X. (2022). Adherence to the 24-Hour Movement Behavior Guidelines and Associations with Depressive Symptoms among College Students. Int. J. Kinesiol. High. Educ..

[19] Muntaner-Mas A., Mora-Gonzalez J., Cabanas-Sánchez V., Pintado L.B., Salmon J., Hillman C.H., Esteban-Cornejo I. (2022). Prospective associations between physical fitness and executive function in adolescents: The UP&DOWN study. Psychol. Sport Exerc..

[20] Lemes V.A., Gaya A.R., Sadarangani K.P., Aguilar-Farias N., Rodríguez-Rodríguez F., Martins C.M.L., Fochesatto C.F., Cristi-Montero C. (2021). Physical Fitness Plays a Crucial Mediator Role in Relationships among Personal, Social, and Lifestyle Factors with Adolescents’ Cognitive Performance in a Structural Equation Model. The Cogni-Action Project. Front. Pediatr..

[21] Bao R., Leahy A.A., Lubans D.R., Diallo T.M.O., Beauchamp M.R., Smith J.J., Hillman C.H., Wade L. (2024). Mediators of the As-sociation between Physical Activity and Executive Functions in Primary School Children. J. Sports Sci..

[22] Marrero-Rivera J.P., Sobkowiak O., Jenkins A.S., Bagnato S.J., Kline C.E., Gordon B.D., Taverno Ross S.E. (2024). The relationship Between Physical Activity, Physical Fitness, Cognition, and Academic Outcomes in School-aged Latino Children: A Scoping Review. Children.

[23] Eccles J.S., Adler T.F., Futterman R., Goff S.B., Kaczala C.M., Meece J., Midgley C., Spence J.T. (1983). Expectancies, Values, and Academic Behaviors. Achievement and Achievement Motivation.

[24] Eccles J.S., Wigfield A. (1995). In the Mind of the Actor: The Structure of Adolescents’ Achievement task valus and Expectancy-related beliefs. Pers. Soc. Psychol. Bull..

[25] Wigfield A., Eccles J.S. (2000). Expectancy-Value Theory of Achievement Motivation. Contemp. Educ. Psychol..

[26] Chen S., Chen A., Sun H., Zhu X. (2013). Physical Activity and Fitness Knowledge Learning in Physical Education: Seeking a Common Ground. Eur. Phys. Educ. Rev..

[27] Gu X., Solmon M.A., Zhang T. (2012). Using Expectancy-Value Model to Examine Students’ Physical Activity Engagement and Cardiovascular Fitness in Physical Education. Int. J. Sport. Psychol..

[28] Shang C., Moss A.C., Chen A. (2023). The Expectancy-Value Theory: A Meta-Analysis of Its Application in Physical Education. J. Sport Health Sci..

[29] Chen H., Sun H., Dai J., Griffin M. (2017). Relationships among Middle School Students’ Expectancy Beliefs, Task Values, and Health-Related Fitness Performance. J. Teach. Phys. Educ..

[30] Gu X., Solmon M.A. (2015). Motivational Processes in Children’s Physical Activity and Health-Related Quality of Life. Phys. Educ. Sport Pedagog..

[31] Gu X., Chen S., Zhang X. (2018). Young Hispanic and Non-Hispanic Children’s Fundamental Motor Competence and Physical Activity Behaviors. J. Mot. Learn. Dev..

[32] Gu X., Zhang T., Chu A., Wang J., Zhang X., Nelson L., Brown K. (2019). Exploring Racial Disparities in Physical Activity and Quality of Life through an Expectancy-Value Perspective. J. Racial Ethn. Health Disparities.

[33] Gråstén A., Watt A., Liukkonen J., Jaakkola T. (2017). Effects of School-Based Physical Activity Program on Students’ Moder-ate-to-Vigorous Physical Activity and Perceptions of Physical Competence. J. Phys. Act. Health.

[34] Gu X., Fu Y., Chen W., Tamplain M.P., Zhang T., Wang J. (2021). A Causal Pathway of Physical Activity to Motor Competence in Childhood: Evidence from a School-Based Intervention. J. Sports Sci..

[35] U.S. Department of Health and Human Services (2012). The 2012 HHS Poverty Guidelines. Fed. Regist..

[36] Plowman S.A., Meredith M.D. (2013). FITNESSGRAM^®^/ACTIVITYGRAM^®^ Reference Guide.

[37] Guy S.C., Isquith P.K., Gioia G.A. (2004). Behavior Rating Inventory of Executive Function–Self-Report Version (BRIEF-SR).

[38] Xiang P., McBride R., Guan J., Solmon M. (2023). Children’s motivation in elementary physical education: An expectancy-value model of achievement choice. Res. Q. Exerc. Sport.

[39] Zhu X., Sun H., Chen A., Ennis C. (2012). Measurement Invariance of Expectancy-Value Questionnaire in Physical Education. Meas. Phys. Educ. Exerc. Sci..

[40] Mahar M.T., Guerieri A.M., Hanna M.S., Kemble C.D. (2011). Estimation of Aerobic Fitness from 20-m Multistage Shuttle Run Test Performance. Am. J. Prev. Med..

[41] Meredith M.D., Welk G.J. (2013). FITNESSGRAM^®^/ACTIVITYGRAM^®^ Test Administration Manual.

[42] Hinze V., Blakemore S.J., Dalgleish T., Ford T., Mansfield K.L., Ukoumunne O.C., Kuyken W., Montero-Marin J. (2025). The Longitudinal Relationship Between Self-Reported Executive Function and Mental Health in Early Adolescence. JAACAP Open.

[43] Pablo-Ríos M.V., Navarro-Asencio E., Mateos-Gordo P., García-Gómez R., Porras-Truque C., García Moreno L.M. (2024). Dysexecutive Symptomatology in Everyday Functioning and Academic Achievement in Adolescents. Front. Psychol..

[44] Bennett D.A. (2001). How Can I Deal with Missing Data in My Study?. Aust. N. Z. J. Public Health.

[45] Eccles J.S., Wigfield A. (2020). From expectancy-value theory to situated expectancy-value theory: A developmental, social cognitive, and sociocultural perspective on motivation. Contemp. Educ. Psychol..

[46] Tang X., Lee H.R., Wan S., Gaspard H., Salmela-Aro K. (2022). Situating expectancies and subjective task values across grade levels, domains, and countries: A network approach. Aera Open.

[47] Kline R.B. (2011). Principles and Practice of Structural Equation Modeling.

[48] Hu L., Bentler P.M. (1999). Cutoff Criteria for Fit Indexes in Covariance Structure Analysis: Conventional Criteria versus New Alternatives. Struct. Equ. Model..

[49] Browne M.W., Cudeck R. (1992). Alternative ways of assessing model fit. Sociol. Methods Res..

[50] Chaddock L., Hillman C.H., Pontifex M.B., Johnson C.R., Raine L.B., Kramer A.F. (2012). Childhood Aerobic Fitness Predicts Cognitive Performance One Year Later. J. Sports Sci..

[51] Raine L.B., Kao S.C., Pindus D., Westfall D.R., Shigeta T.T., Logan N., Cadenas-Sanchez C., Li J., Drollette E.S., Pontifex M.B. (2018). A Large-Scale Reanalysis of Childhood Fitness and Inhibitory Control. J. Cogn. Enhanc..

[52] Caspersen C.J., Powell K.E., Christenson G.M. (1985). Physical activity, exercise, and physical fitness: Definitions and distinctions for health-related research. Public Health Rep..

[53] Ortega F.B., Ruiz J.R., Castillo M.J., Sjöström M. (2008). Physical fitness in childhood and adolescence: A powerful marker of health. Int. J. Obes..

[54] Britton U., Issartel J., Fahey G., Conyngham G., Belton S. (2020). What is health-related fitness? Investigating the underlying factor structure of fitness in youth. Eur. Phys. Educ. Rev..

[55] Wiguna T., Minayati K., Kaligis F., Teh S.D., Krishnandita M., Meriem Annisa Fitri N., Ismail R.I., Fasha A.H., Steven Bahana R. (2024). Using the Indonesian Computer-Based Game Prototype as a Computer-Based Game Inventory for Executive Function in Children and Adolescents: A Confirmatory Factor Analysis and Concurrent Validity Study. Assessment.

